# Self-reported efficacy of complementary and alternative medicine in chronic headache subjects in the general population

**DOI:** 10.1186/1129-2377-14-S1-P23

**Published:** 2013-02-21

**Authors:** ES Kristoffersen, K Aaseth, RB Grande, C Lundqvist, MB Russell

**Affiliations:** 1 Head and Neck Research Group, Research Centre, Akershus University Hospital, Lørenskog, Norway

## Background

Chronic headache is associated with disability and high utilisation of health care including complementary and alternative medicine (CAM). We have previously shown that 62% of primary and 73% of secondary chronic headache sufferers from the general population have tried CAM for their headache but the efficacy of this use as treatment for chronic headache is not known.

## Methods

An age and gender stratified cross-sectional epidemiological survey included 30,000 persons aged 30-44 years. Respondents with self-reported chronic headache were interviewed. The International Classification of Headache Disorders was used. Participants with primary or secondary chronic headache were asked about previous use of CAM and efficacy for their headache. Modalities of CAM queried were acupuncture, chiropractic, homeopathy, naprapath, physiotherapy, psychologist, and psychomotor physiotherapy.

## Results

The questionnaire response rate was 71%, the interview participation rate 74%. Of 405 subjects with primary chronic headache, 253(62%) had used CAM for their headache. Of 113 subjects with secondary chronic headache, 82(73%) had used CAM. The self-reported efficacy ranged from 15-35% and 6-38%, respectively for primary and secondary chronic headaches depending on CAM modality being used. Generally, there were no significant differences in self-reported efficacy of CAM depending on gender, co-occurrence of migraine, medication overuse or physician contact. Of the most commonly used CAM modalities, subjects with primary chronic headache reported greatest efficacy of psychomotor physiotherapy(35%) > chiropractic(26%) > physiotherapy(25%). Of the most commonly used CAM modalities, subjects with secondary chronic headache reported greatest efficacy of physiotherapy (38%) = chiropractic(38%) > acupuncture(32%).

## Conclusion

Self-reported efficacy of different CAM modalities in chronic headache subjects from the general population is modest.

